# Leveraging Dental Biotechnology for Population Oral Health: Innovations in Prevention, Diagnosis, and Treatment

**DOI:** 10.3390/ijms262211188

**Published:** 2025-11-19

**Authors:** Omer Faruk Sonmez, Thuto Serufe Makara, Raman Bedi

**Affiliations:** 1 School of Medicine and Population Health, University of Sheffield, Sheffield S10 2TN, UK; 2 Vocational School of Health Services, Oral Health Programme, Kırıkkale University, 71450 Kırıkkale, Türkiye; 3 World Federation of Public Health Associations (WFPHA), 1202 Geneva, Switzerland; 4 Faculty of Dentistry, Oral and Craniofacial Sciences, King’s College London, London WC2R 2ND, UK

**Keywords:** dental biotechnology, salivary diagnostics, biomaterials, regenerative dentistry, vaccines

## Abstract

Biotechnology is reshaping dental public health by providing new tools for prevention, diagnosis, and treatment of oral diseases at scale. Salivary biomarkers enable non-invasive, early detection of caries, periodontitis, and oral cancer. Tissue engineering and regenerative approaches, driven by stem cell signaling and bioactive scaffolds, offer biologically integrated repair. Genomic discoveries now allow polygenic risk profiling to complement social determinants in identifying vulnerable groups, while novel biomaterials, probiotics, and vaccine research expand options for sustainable caries prevention. These innovations are underpinned by molecular mechanisms such as inflammatory signaling, stem cell differentiation pathways, and antimicrobial activity. Their translation into public health practice requires attention to affordability, regulation, equity, and workforce integration. Harnessed effectively, biotechnology can help shift oral health systems toward more preventive and equitable models of care.

## 1. Introduction

Oral diseases such as dental caries, periodontal disease, and oral cancer remain among the most widespread chronic conditions globally, affecting nearly half of the world’s population. The World Health Organization (WHO) estimates that 3.7 billion people live with untreated oral disease, with the heaviest burden concentrated in low- and middle-income countries [1]. Traditional public health measures including fluoride programs, community outreach, and expansion of the dental workforce have produced measurable gains, yet they have not succeeded in reducing the inequities that define the global oral health crisis.

Biotechnology offers complementary and potentially transformative approaches to oral health. Advances in salivary diagnostics, regenerative and restorative biotechnology, genomics, and antimicrobial dental materials are shifting the field from reactive treatment to proactive, preventive, and personalized strategies [2]. At the molecular level, these innovations harness specific biological processes such as nucleic acid hybridization for biomarker detection, Wnt/β-catenin signaling for pulp regeneration, or reactive oxygen species (ROS) generation for antimicrobial activity to address the fundamental drivers of oral disease. Importantly, biotechnology can extend beyond the clinic and integrate into population-level strategies, opening new possibilities for universal health coverage and precision public health.

This article review aims to (i) describe some promising biotechnological applications in dental public health, examining both the molecular mechanisms underlying these innovations; (ii) critically appraise the strength of evidence and translational reediness; and (iii) outline their potential role in advancing equitable oral health systems at population level.

## 2. Results

### 2.1. Population Diagnostics and Surveillance

Saliva is emerging as a powerful diagnostic fluid because it contains a wide array of molecular markers including DNA, RNA, proteins, metabolites, and microbiome-derived signatures that mirror both oral and systemic health. At the molecular level, inflammatory and immune-related transcripts such as *IL-1β* and *TNF-α*, as well as microRNAs including *miR-21* and *miR-31*, are sensitive indicators of early inflammatory responses and malignant transformation [3]. Tissue breakdown in periodontal disease can be detected through elevated salivary levels of matrix metalloproteinase-8 (MMP-8), while epigenetic alterations, such as hypermethylation in tumor suppressor genes like *p16^INK4a^* and *MGMT*, provide early warning signals for oral cancer [4]. The detection of these biomarkers in point-of-care devices is achieved through technologies such as antigen–antibody binding, nucleic acid hybridization, and electrochemical or optical signal transduction, often miniaturized into lab-on-a-chip formats or integrated biosensors [5]. These approaches allow real-time analysis with high sensitivity and specificity, without the need for invasive sampling or complex laboratory infrastructure. Table 1 below summarizes the diagnostic biomarkers.

Biosensors are compact devices that turn a biological interaction into a readable result. They pair a capture element that recognizes a target molecule with a component that converts that recognition into an electrical or optical signal. Because they can be portable and inexpensive, they are well suited to screening in schools, primary-care clinics, and mobile units. Saliva is a particularly good sample for this purpose: collection is non-invasive, can be repeated as often as needed, and the fluid carries proteins, nucleic acids, metabolites, and other constituents that mirror disease processes [14].

Most oral-health biosensors today use electrochemical or optical readouts. Electrochemical formats register tiny changes in current or voltage when a target binds at the sensor surface and can reach very low detection limits in saliva including reported examples include interleukin-1 in the femtogram-per-milliliter range, hypoxia-inducible factor-1 alpha in the picogram-per-milliliter range with shorter processing time than standard laboratory methods, and nucleic-acid targets such as ORAOV1 at femtomolar concentrations. Silicon-nanowire arrays have also measured inflammatory proteins such as tumor necrosis factor and interleukin-8 at 100 femtograms per milliliter in saliva. Optical formats detect changes in light and lend themselves to simple, immediate readouts; demonstrations include fluorescent immunosensors for Cyfra21-1 in clinical saliva and microfluidic chips that quantify combinations such as interleukin-8, interleukin-1, and matrix metalloproteinase-8 in the low picogram-per-milliliter range. Nanomaterials can sharpen performance and make it easier to test more than one marker at a time. The addition of three-dimensional carbon-nanotube structures has been shown to increase sensor sensitivity by about twenty-fold compared with a sandwich enzyme assay, and upconversion nanoparticle probes have been used to report matrix metalloproteinase-2 activity through a visible change in fluorescence. More broadly, researchers emphasize that a single biomarker is rarely enough for early lesions or potentially malignant disorders; practical screening will benefit from platforms designed to combine several targets in one run [14].

Gingival crevicular fluid is a distinct exudate sampled from the gingival sulcus, located at the site of periodontal inflammation rather than being a composite oral fluid like whole saliva. Its molecular composition reflects local tissue breakdown and neutrophil-driven host responses, yielding higher specificity for current periodontal activity than saliva. Enzymes and inflammatory mediators measured in GCF including activated matrix metalloproteinase-8, MMP-9, interleukin-1β, and related proteolytic by-products track pocket-level collagen degradation and correlate with probing depth, bleeding on probing, and radiographic or clinical attachment changes [9].

**Figure 1:**
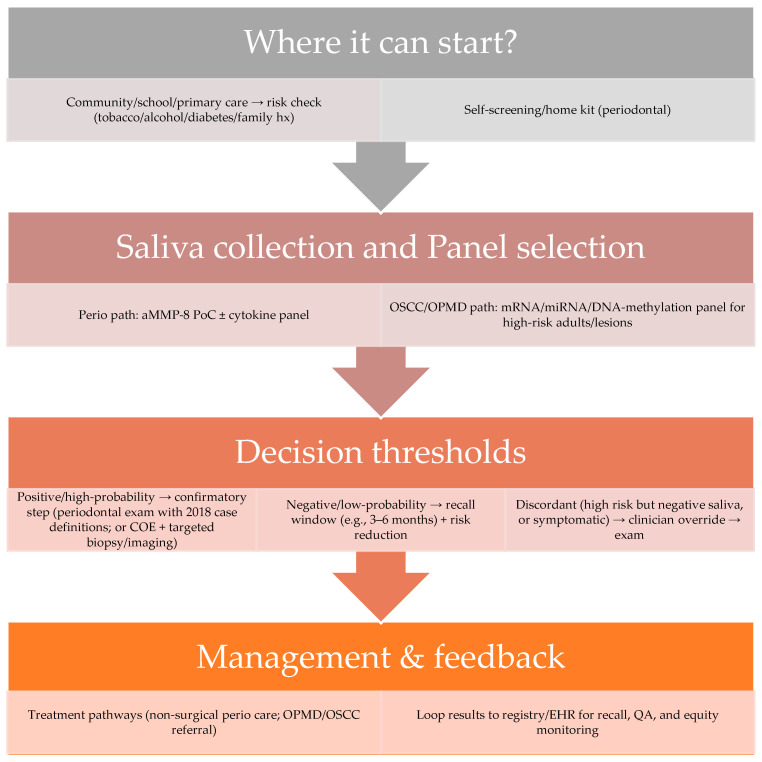
Diagnostic pathway showing integration of salivary biomarkers testing into community and clinical oral health systems. Abbreviations: aMMP-8 = active matrix metalloproteinase-8; PoC = point of care; COE = clinical oral examination; OSCC = oral squamous cell carcinoma; OPMD = oral potentially malignant disorder; mRNA = messenger RNA; miRNA = microRNA; EHR = electronic health record; QA = quality assurance.

From a public health perspective, salivary diagnostics has the potential to shift population oral health strategies from late-stage treatment toward early detection and targeted intervention. The non-invasive nature of saliva collection makes it especially suitable for community-based screening, including in schools, rural outreach programs, and mobile health units. However, while the per-test cost could become low with large-scale manufacturing, the initial investment in devices, reagents, and training remains a significant barrier in low-resource settings. Moreover, many salivary biomarker panels are still in the validation stage, with regulatory approvals and large-scale field trials needed before they can be reliably integrated into national oral health systems. Equitable implementation will depend on ensuring portability, affordability, and culturally appropriate deployment, alongside workforce training and robust quality control systems. Figure 1 highlights the diognastic pathways of salivary biomarkers’ integration into health systems.

Portable biosensors are promising because they can be used in schools and mobile clinics and may test several markers at once, yet most platforms are still at an early stage. A sensible program would begin with low-complexity inflammatory or protein tests inside a clear pathway: set thresholds, confirm findings with an exam, and schedule recall. Build in quality assurance and simple data reporting so teams can track follow-up and equity. To prevent gaps from widening, keep per-test costs low through procurement, train non-specialist staff to collect and run tests, and link results to existing school and primary care services.

### 2.2. Regenerative and Conservative Repair

Regenerative dentistry now draws on an advanced understanding of cellular signaling and biomaterial science to restore dental structures through tissue engineering and biologically driven repair. Central to this process are dental pulp stem cells (DPSCs), which can be obtained from extracted or exfoliated teeth and possess the capacity to differentiate into odontoblasts, osteoblasts, chondrocytes, and other cell types. Their regenerative potential is orchestrated by signaling pathways such as Wnt/β-catenin, which drives odontoblast-like differentiation and tertiary dentin formation through the regulation of Axin2 and other downstream targets [15]. Activation of this pathway with small molecules, including GSK3β inhibitors, has been shown to enhance reparative dentinogenesis and pulp healing. Bone morphogenetic proteins, particularly BMP7, act in concert with Wnt signaling to stimulate the regeneration of pulp, periodontal ligament, alveolar bone, and craniofacial tissues [16]. Enamel matrix derivatives, such as the porcine-derived Emdogain, add another regenerative dimension by mimicking the natural developmental processes of cementogenesis. Rich in amelogenin and associated proteins, these derivatives promote the proliferation and differentiation of periodontal ligament fibroblasts, encourage the deposition of cementum and bone, and suppress unwanted epithelial proliferation, enabling more complete periodontal regeneration. Scaffold technology further enhances regenerative outcomes; hydrogel-based constructs seeded with DPSCs or their extracellular vesicles support osteogenesis and alveolar bone regeneration through direct cellular activity and paracrine signaling [17]. Recent advances in 3D bioprinting and bioactive biomaterials are bringing the prospect of custom-fitted scaffolds for pulp–dentin complex reconstruction closer to clinical reality, although much of this work remains in preclinical development.

From a public health standpoint, the promise of regenerative biotechnology lies in its ability to provide durable, biologically integrated solutions that could reduce the need for repeated restorative procedures and their associated costs over a patient’s lifetime. In theory, these approaches could be adapted for use in community-based clinics, allowing trained dental practitioners to restore pulp vitality, periodontal attachment, or lost craniofacial structures even in underserved populations. However, significant challenges must be addressed before widespread implementation is feasible. The infrastructure required for stem cell culture, scaffold preparation, and sterile biomaterial handling is often absent in low-resource settings. Ethical and regulatory frameworks for stem cell-based therapies remain complex and variable across jurisdictions, and long-term safety data are still emerging. Moreover, despite encouraging early results from clinical trials involving enamel matrix derivatives and DPSC-mediated regeneration, large-scale, population-focused studies are scarce, leaving questions about cost-effectiveness, scalability, and cultural acceptance unresolved. Realizing the potential of regenerative biotechnology in public dental health will require coordinated investment in infrastructure, workforce training, regulatory alignment, and the development of simplified, lower-cost delivery systems that can translate laboratory advances into practical tools for equitable care.

### 2.3. Genomic Targeting and Risk Stratification

Across common oral diseases, the underlying biology is decisively polygenic: dozens to hundreds of common variants of small effect, layered with rare variants of larger effect, shape susceptibility while interacting with environment and behavior. Genome-wide association studies (GWAS) continue to expand this map for dental caries and periodontitis, most recently identifying new caries loci (e.g., intronic variants in GLIS3 and SIGLEC5) and refining shared biology between clinical and self-reported phenotypes, while multi-ancestry analyses underscore heterogeneous architecture across populations [18]. Polygenic risk scores (PRS) aggregate small effects into an individual-level liability index, a tool increasingly evaluated alongside epigenetic and transcriptomic markers to capture gene–environment interplay in the oral cavity. In caries, PRS derived from large GWAS now predict prevalent and incident disease, albeit modestly, when tested prospectively in adult biobanks, suggesting a role as one component of risk stratification rather than a stand-alone screen with reported area under the receiver operating characteristic curve (AUC) typically in the range of about 0.60–0.68 [19]. For periodontitis, recent reviews and new GWAS highlight biologic plausibility but also emphasize that current PRS performance and portability remain limited without larger, more diverse discovery datasets and harmonized clinical phenotypes [20]. In craniofacial anomalies, polygenic burden modifies the penetrance of high-impact variants (e.g., PDGFRA) and helps localize effector cell types for non-syndromic cleft lip/palate (nsCL/P), providing a clearer mechanistic bridge from common variant load to disrupted facial morphogenesis [21]. Molecular insights reposition genomics away from single-gene explanations toward population-calibrated, polygenic indices that can be paired with modifiable exposures to anticipate disease trajectories [22].

For public health planning, the practical value of genomics is not in predicting destiny but in targeting prevention where baseline risk is higher, and benefits accrue sooner. In caries-prone communities, a PRS-anchored “precision prevention” workflow can stratify cohorts at school or primary-care entry, then intensify low-cost interventions such as high-fluoride toothpaste, quarterly varnish and fissure sealants, motivational interviewing, and dietary sugar counseling; while reserving more intensive follow-up for the top risk decile; early adult data indicate that PRS can flag individuals with higher caries experience over time, supporting such tiered pathways when combined with clinical and social determinants. In periodontitis, near-term use is more circumscribed: PRS can be explored as an adjunct to classic risk scores (smoking, diabetes, plaque indices) to prioritize periodontal screening intervals, but translation will depend on larger multi-ancestry GWAS, standardized case definitions, and careful evaluation of net reclassification beyond traditional predictors. For orofacial clefts, family-based counseling that integrates common-variant polygenic load with rare variant testing can refine recurrence counseling and guide antenatal surveillance and peri-natal care planning in high-incidence regions. Across these use-cases, implementation must foreground equity: discovery datasets remain Eurocentric, and PRS accuracy drops when applied across ancestries; therefore, any deployment should pair local re-weighting or re-training with community-based consent, clear communication of probabilistic risk, and strict safeguards against stigmatization or insurance misuse. When embedded in existing prevention platforms, school dental programs, maternal and child health visits, mobile clinics; polygenic tools can help focus scarce resources rather than replace proven measures, aligning genomics with population benefit rather than boutique personalization. Identifying high-risk individuals or groups on a genomic basis would require widespread or universal genetic screening, an approach that is prohibitively expensive, logistically complex, and ethically fraught. At present, it is far more practical for public health systems to prioritize preventive interventions based on socioeconomic vulnerability, where the burden of oral disease is well documented, rather than attempting to stratify populations by genetic risk [23].

### 2.4. Antimicrobial and Preventive Biomaterials

Antimicrobial agents in dentistry aim to suppress biofilm formation where disease tends to recur at restoration margins and implant surfaces. Two broad strategies exist. First, non-leaching, contact-killing surfaces such as quaternary ammonium that are functional resins and antimicrobial-peptide-grafted or nano-textured titanium can inhibit early colonization; critical reviews show strong in vitro/preclinical effects but emphasize variable durability and limited clinical endpoints so far [24,25,26]. TiO_2_-nanotube and related nanotopographies reduce bacterial adhesion and can be coupled to photocatalysis, yet reviewers repeatedly note the need for translational and long-term human data [27,28,29].

Controlled-release approaches deliver antimicrobial activity from within the material: examples include chlorhexidine-modified glass ionomers (sustained CHX release reported up to ~14 months in lab studies), nitric-oxide-releasing coatings built on SNAP chemistries, and nanoparticle/ion-releasing fillers (e.g., Ag, ZnO, bioactive glasses) [30,31]. Across both camps, the main trade-offs are dose-dependent cytotoxicity, possible color/translucency changes, and mechanical compromises at higher loadings; contemporary reviews urge cautious interpretation and standardized testing [24,32]. What would be truly groundbreaking is high-quality, multicenter evidence that a surface or restorative system reduces peri-implantitis or secondary caries over 3–5 years without harming osseointegration or restoration longevity which is an outcome current consensus/overviews say remains to be conclusively shown [33].

At a population level, innovation will rise or fall on implementation enablers and system constraints. Facilitators include alignment with the WHO Global Oral Health Action Plan and universal health coverage packages (creates policy pull), fit with HTA/value-for-money frameworks (lets payers compare against proven preventives like sealants/fluoride), and procurement models that can scale training and quality assurance [34]. that materials reduce disease or retreatments at scale and credible budget-impact/cost-effectiveness relative to existing programs will be persuasive; for context, school sealant programs often pass cost-effectiveness thresholds, with labor/time the major cost driver [35]. Barriers include regulatory complexity (e.g., EU MDR tightening evidence and post-market surveillance for novel biomaterials), methodological gaps for evaluating device-like innovations, and the need to monitor safety/externalities such as environmental release of nanosilver [36,37].

Recent years have seen transformative strides in dental biomaterials, as scientists harness nanotechnology, antimicrobial peptides, and intelligent release systems to develop surfaces and materials that actively combat cariogenic microbes and biofilms.

Silver nanoparticles (AgNPs) have emerged as one of the most promising antimicrobial additions. Their nanoscale size and high surface area enable generation of reactive oxygen species (ROS), membrane disruption, and bacterial DNA damage. Reviews consistently show that AgNP incorporation into composites, adhesives, and dental cement significantly boosts antibacterial capacity and inhibits plaque-forming bacteria, including *Streptococcus mutans* [38]. TiO_2_ and zinc oxide nanoparticles echo these antimicrobial properties, with added photocatalytic benefits for materials that activate under light [39]. However, systematic reviews also highlight a trade-off: while antimicrobial efficacy improves, mechanical properties such as strength or color stability may suffer, necessitating careful optimization of nanoparticle type, concentration, and integration method [40].

Beyond nanoparticles, peptide-based coatings represent a biologically elegant strategy. Antimicrobial peptides like GL13K, when chemoselectively bound to titanium implant surfaces, impede bacterial colonization and hold potential for envelope-safe coatings applicable in restorative or prosthetic dentistry [41].

Probiotic and postbiotic therapies offer a fundamentally different mode of microbial control. Instead of killing pathogenic species, these strategies restore a balanced oral microbiome. Strains such as *Lactobacillus acidophilus*, *L. reuteri*, and *Bifidobacterium longum* have demonstrated inhibition of *S. mutans* biofilm formation, downregulation of its glucosyltransferases, and better ecological stability [42]. Though promising, these interventions have limited clinical validation and must complement and not replace established preventive measures such as fluoride.

Molecular mechanisms are at the heart of these advances. Silver and metal nanoparticles wield ROS-mediated, multi-target antibacterial action. Antimicrobial peptides disrupt cell membranes or signal immune modulation. Probiotics and postbiotics act through competitive exclusion, immune modulation, and metabolic interference. Vaccine and replacement strategies focus on either immunizing against, or displacing, cariogenic species [43].

In the context of public dental health, these innovations are promising. Integrating AgNP-embedded sealants or peptide-coated liners into routine restorative workflows could reduce secondary caries and enhance longevity of restorations. Probiotics or postbiotics might become school-based oral health supplements to support anticariogenic microbial ecology. Self-assembling peptides like P11-4 may be deployed via minimally invasive, community-based enamel repair clinics especially if formatted into affordable, easy-use products.

Alongside their antimicrobial benefits, it is equally important to consider the long-term safety and environmental impact of these emerging materials. Biocompatibility assessments should extend beyond short-term cytotoxicity to include possible genotoxic, immunologic, and chronic-exposure effects, particularly in nano-based formulations. Recent studies on nanoparticle leaching, such as the release of nano silver into wastewater, highlight the need for life cycle monitoring and environmentally responsible disposal [44]. Regulatory frameworks are also evolving, with both the European Union Medical Device Regulation (EU MDR) and the U.S. FDA emphasizing materiovigilance, post-market surveillance, and transparent reporting of any adverse or ecological effects associated with dental materials. Embedding these safeguards into product design, clinical protocols, and procurement systems will help ensure that new technologies remain safe for patients and sustainable for the environment.

### 2.5. Vaccinology and Microbiome Engineering

An emerging frontier is the development of anti-caries vaccines and designer microbiome interventions. Decades of vaccine research targeting *S. mutans* have yet to culminate in a licensed human vaccine. However, innovative paths are evolving genetically modified strains like BCS3-L1 (a “replacement therapy”) have undergone initial human testing, while companies are exploring self-assembling peptides (like P11-4) that engineer tooth mineralization when combined with saliva, promoting enamel regeneration. Though not yet mainstream or proven at scale, these efforts offer a glimpse into biologically proactive prevention [45].

Recent immunological innovations in periodontal care are exploring mucosal vaccines that target key dysbiotic microbes and the host–pathogen interface at the periodontal attachment rather than solely relying on restorative or mechanical interventions. For example, animal models using intranasal delivery of trivalent vaccines directed against *Porphyromonas gingivalis*, *Tannerella forsythia* and *Fusobacterium nucleatum* have demonstrated significant reductions in alveolar bone loss as measured from the cementoenamel junction to the alveolar crest (CEJ-ABC). The CEJ serves as a reproducible anatomical landmark in periodontal research and allows quantification of vaccine impact on attachment loss trajectories. In practical terms for public-health programs, these vaccines open a future pathway in which immune priming could reduce the rate of attachment loss, lessen the need for surgical intervention, and shift the paradigm from reactive treatment to pre-emptive periodontal care [46,47].

Although routine immunization programs are not designed for teeth and gums, they already influence oral health in meaningful ways and point toward what a true oral-disease vaccine might look like. The clearest current example is the human papillomavirus (HPV) vaccine [48]. By preventing infection with high-risk HPV types, it lowers the pool of oral HPV in the population and is expected to reduce oropharyngeal cancers over time; national guidance now frames HPV vaccination as cancer prevention for all adolescents, not just girls, with accumulating evidence of protection against oral HPV infection and downstream head-and-neck cancers [49]. At the same time, concerns about vaccine-related problems inside the mouth deserve an honest summary. Across surveillance systems and reviews, oral side effects after common vaccines that was reported were taste disturbance, mouth ulcers, or transient mucosal irritation but they are uncommon, generally short-lived, and far outweighed by the benefits of preventing serious infections; this pattern has been documented for COVID-19 vaccines and appears similar to what is seen after seasonal influenza vaccination [50].

Looking ahead to vaccines that directly target oral diseases, caries is the closest candidate because it has well-defined microbial drivers and surface virulence factors that can be recognized by the immune system. Streptococcus mutans uses adhesins and enzymes to stick to enamel and build acid-producing biofilms; decades of laboratory work and animal studies show that immunization against these proteins can reduce colonization and caries in preclinical models. The most frequently studied targets include the antigen I/II adhesin (also called PAc), glucosyltransferases that build sticky glucans, and glucan-binding proteins; several strategies combine these antigens into a single construct to improve coverage. The central idea is to trigger strong secretory immunoglobulin A in saliva so that bacteria are blocked from binding and building biofilm on tooth surfaces. Despite progress, no caries vaccine is licensed for human use yet; recent scoping and narrative reviews conclude that the field remains preclinical or early-phase, with promising immunogenicity but many practical questions about durability, breadth of protection across strains, and delivery route [51].

Periodontal disease presents a tougher vaccine problem because the damage is driven by a complex community of organisms and a dysregulated host response rather than a single pathogen. Still, there are plausible targets. For *Porphyromonas gingivalis*, candidates include the fimbriae used for attachment and the proteases (“gingipains”) that help the organism disrupt tissue; experimental vaccines against these components can dampen inflammation and tissue breakdown in animals, and early human studies suggest that even passive immunization with monoclonal antibodies can hinder recolonization. Any successful approach will likely need to raise protective antibodies on the oral mucosa, so routes like nasal, sublingual, or buccal dosing are under active discussion. As with caries, there is no licensed periodontal vaccine, and reviews emphasize the need to cover multiple antigens and to show real clinical benefit on attachment loss and tooth retention, not just antibody levels [52,53].

If and when these vaccines arrive, what forms are most likely? Classical protein subunit designs (purified bacterial proteins with an adjuvant) remain the workhorse in dental vaccine research because they are well understood and relatively safe. DNA plasmid vaccines and peptide vaccines have also been explored in animals. Newer platforms are attractive for oral conditions because they can be built and updated quickly: messenger RNA (mRNA) packaged in lipid nanoparticles is a leading example from general vaccinology, and intranasal mRNA delivery has already produced strong mucosal immune responses in animals which is a property that could be crucial for bathing teeth and periodontal tissues with protective antibodies. That said, current mRNA work for dentistry is still preclinical, and no human trials of an mRNA caries or periodontitis vaccine have been completed [54,55].

Beyond scientific feasibility, the development of vaccines for oral diseases also brings important ethical, regulatory, and social considerations. Mucosal or oral vaccines, in particular, must meet high standards of safety and quality while taking into account public perceptions of new delivery routes. Building trust will depend on transparent communication, informed consent, and culturally sensitive engagement with communities. As regulatory pathways for dental vaccines are still taking shape, early collaboration with health authorities will be essential to ensure ethical oversight, equitable access, and long-term safety as these innovations move toward clinical use.

## 3. Discussion

Biotechnology has matured to a point where translation into public oral health is realistic, but success will be decided by implementation rather than invention.

Non-invasive salivary diagnostics and portable biosensors can extend screening beyond dental surgeries into schools, primary care, and mobile programs. To avoid false assurance or over-referral, programs should adopt multi-test pathways with clear thresholds, confirmatory clinical examination, and quality assurance that includes calibration, proficiency testing, and external controls. Data flows must be designed for action: secure, interoperable reporting into electronic records that support recall, audit, and equity monitoring.

Regenerative and restorative innovations promise durable, biologically integrated care, yet their infrastructure demands, regulatory complexity, and cost structures make near-term community deployment challenging. A pragmatic route is to prioritize adjuncts already used in practice such as enamel matrix derivatives and bioactive materials while building evidence for cell-based and 3-dimensional bioprinting approaches through multicenter trials that report outcomes relevant to populations: retreatment rates, tooth survival, function, and quality of life.

In parallel, antimicrobial materials and coatings should be evaluated with standardized methods that balance antibacterial performance with mechanical integrity, esthetics, biocompatibility, and environmental safety.

Genomic insights can complement, not replace, established prevention. Polygenic risk tools have modest predictive value and reduced portability across ancestries; their ethical use requires local validation, transparent communication of probabilistic risk, and strict safeguards against misuse.

In public health planning, socioeconomic vulnerability remains the most practical and just basis for intensifying preventive care, including fluoride, sealants, dietary counseling, and tobacco cessation. Probiotic and postbiotic strategies, as well as vaccine research aimed at cariogenic and periodontal pathogens, are promising but should be integrated only as adjuncts once efficacy, durability, and delivery routes are established ideally mucosal for oral targets.

Across domains, the same enabling conditions recur fit with universal health coverage packages; procurement models that ensure reliable supply and maintenance; training that allows task-sharing with non-specialist providers; and evaluation through health technology assessment, budget-impact analyses, and materiovigilance for devices and materials.

Equity should anchor every step from study recruitment and site selection to pricing, language-appropriate consent, and community engagement so that innovations narrow, rather than widen, oral health gaps.

### Translating Biotechnological Interventions into Population-Level Health Gains

Turning promising dental biotechnologies into public programs benefits from a clear sequence of questions that have already been well defined in public health and implementation science. Below we outline a pragmatic pathway that adapts widely used frameworks to oral health delivery.

**(1)**  **Is the problem suitable for programmatic action?** Initiation of a population program requires evidence of substantial disease burden and a plausible, modifiable pathway to improved outcomes through primary prevention, early management, or treatment. Adoption should be supported by effectiveness data from comparable settings, a favorable benefit-to-harm balance, and feasibility within existing workforce, infrastructure, and supply chains. Equity and affordability must be specified in advance, including strategies to reach priority groups and to prevent exclusion. The operational pathway should be defined end to end, covering eligibility, consent, delivery, follow up, adverse event management, and routine data capture, so that enrolment reliably translates into measurable health gain [56,57,58].
**(2)**  **What does a public-health–ready product look like?** Population use requires a target product profile that covers both the intervention and its delivery. Specify intended use, target population, performance thresholds, safety requirements, contraindications, and the minimum evidence for adoption. For diagnostics and triage tools, benchmarks can draw on WHO ASSURED that include connectivity and simple specimen collection. For preventive and therapeutic products, define expected effect size or noninferiority, dosing, durability of benefit, and acceptable adverse event rates, with clear linkage to existing care pathways [59].
**(3)**  **Is the technology mature enough to leave the lab?** Technology readiness frameworks adapted for health products offer a staged taxonomy from concept through analytical validation, design verification, clinical evaluation, and early use in practice. Applying these stages as explicit decision gates helps align scientists, regulators, and funders on what evidence is required before field work, reduces premature piloting, and clarifies the next study needed to advance maturity. For diagnostics, this typically entails sequential demonstration of analytical performance, clinical validity, and clinical utility. For devices, it includes verification and validation against standards, usability testing in intended settings, and initial safety and performance data prior to wider implementation [60].
**(4)**  **How can evidence be designed for real-world delivery and effective implementation?** Development and evaluation should follow guidance for complex interventions that foregrounds program theory, context, and iterative refinement, with study designs calibrated toward routine care using tools that position trials on the pragmatic to explanatory spectrum so that eligibility, endpoints, and follow up reflect actual services. In parallel, implementation determinants should be assessed systematically to anticipate barriers and enablers across intervention features, inner and outer settings, individuals, and processes. This assessment should inform training, workflow integration in primary care, schools, and mobile units, procurement and maintenance plans, data capture and interoperability, and strategies for fidelity and appropriate adaptation. Embedding mixed-methods process evaluation and cost measurement within trials and pilots links effectiveness with feasibility and acceptability and produces actionable guidance for scale up [61,62,63].
**(5)**  **How will the program demonstrate population impact beyond trial efficacy?** Evaluation extends beyond internal validity to encompass reach, uptake, implementation quality, and durability of effects. Analyses quantify the proportion and representativeness of the eligible population that receives the intervention, document changes in clinical and patient outcomes including unintended effects and their distribution across subgroups and measure organizational and provider uptake with attention to non-adopting settings. Implementation is examined through fidelity to core components, context-appropriate adaptations, resource utilization, and direct and indirect costs. Follow-up assesses maintenance of effects at the individual level and sustained delivery at the organizational level over time [64].
**(6)**  **Can payers and policymakers justify adoption and reimbursement?** Decision making relies on formal health technology assessment that integrates comparative clinical effectiveness, safety, and economic value with organizational, ethical, social, legal, and equity considerations. Adoption is justified when the intervention demonstrates meaningful health gain relative to current practice, affordability within the health budget, operational feasibility in the intended settings, and a net positive impact on equity [36].

**Figure 2:**
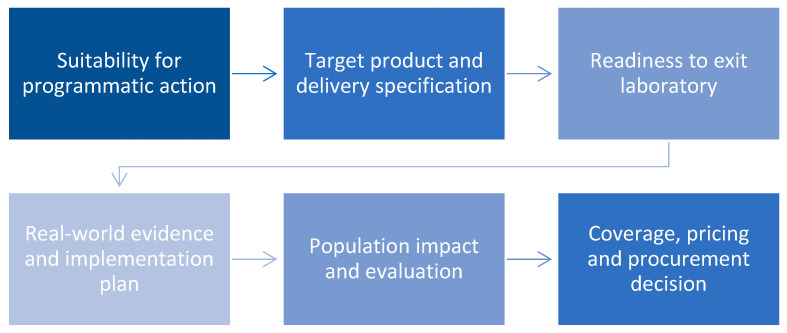
Framework for translating dental biotechnology into population health programs.

As illustrated in Figure 2 cross-cutting safeguards underpin every stage of translation from innovation to population delivery. Ensuring equitable access requires attention to affordability, task sharing, and cultural appropriateness, so that new technologies reduce rather than widen disparities. Workforce development and training are essential to support safe and consistent use of biotechnologies across diverse service settings. Strong data governance, interoperable information systems, and reliable supply chains sustain program quality and accountability. Finally, materiovigilance and environmental monitoring safeguard users and communities by tracking the safety and ecological impact of new materials and devices. Together, these safeguards create the foundation for sustainable and ethically responsible implementation at scale.

Overall, the strength of evidence across biotechnological domains remains variable. Salivary diagnostics and bioactive materials are supported by meta-analyses and early implementation data, indicating moderate-to-high confidence in feasibility for near-term integration. In contrast, regenerative cell-based therapies and genomic tools remain at exploratory or proof-of-concept stages, requiring robust multicenter validation and health-economic evaluation before adoption.

While this review outlines promising directions for dental biotechnology, several limitations should be acknowledged. The strength and maturity of evidence vary considerably across domains. Many regenerative and genomic studies are still at the proof-of-concept stage, and validation of salivary biomarkers remains constrained by small, heterogeneous samples. Most large-scale genomic datasets continue to over-represent populations of European ancestry, limiting the generalizability of current findings. Because this is a broad and fast-moving field, not every relevant study could be cited or explored in depth, and some emerging data may therefore be under-represented. This reflects the pace of discovery rather than deliberate omission, but it reinforces the need for regular evidence updates as the science evolves. These gaps underscore the need for multi-center trials, standardized protocols, and more inclusive research to support translation into diverse real-world settings.

Turning these scientific advances into practical public-health programs will depend on affordability, regulatory alignment, and workforce capability. Success will require coordinated procurement systems, reliable supply chains, and integration with existing primary-care services. Training non-specialist providers, maintaining strong quality-assurance processes, and using interoperable data systems for monitoring and evaluation can all help ensure consistent delivery. Equally important are materiovigilance and environmental safeguards to ensure that new materials and devices remain safe, sustainable, and acceptable for the communities they are designed to serve.

Future research should prioritize pragmatic, multi-center trials that report population-level outcomes such as retreatment rates, tooth survival, and quality of life. Comparative-effectiveness studies are also needed that directly benchmark emerging biotechnologies against established preventive and restorative interventions, ensuring that translation is guided by evidence quality and population benefit rather than technological novelty. Standardized evaluation frameworks for biomaterials including environmental monitoring together with diverse, multi-ancestry genomic validation and equity-focused mucosal vaccine platforms, will be key to supporting safe and inclusive implementation at scale.

## 4. Methods

This narrative review was conducted through structured searches of PubMed, Scopus, and Web of Science databases covering publications from 2015 to 2025. Search terms combined concepts related to dental biotechnology, salivary diagnostics, biomaterials, regenerative dentistry, vaccines, and genomics using Boolean operators. Peer-reviewed articles, systematic reviews, and key preclinical or translational studies were included if they reported molecular mechanisms, clinical applications, or public-health implications of biotechnological innovations relevant to oral health. Exclusion criteria comprised non-English publications, conference abstracts without full text, and studies unrelated to population oral-health contexts. Additional sources, such as WHO and regulatory guidance documents, were incorporated to contextualize implementation and policy relevance.

## 5. Conclusions

Dental biotechnology can reorient oral health systems toward prevention, earlier detection, and biologically conservative care. To translate promise into population benefit, policymakers and practitioners should prioritize technologies that are affordable, simple to operate, validated in diverse communities, and easy to integrate with existing prevention and primary-care platforms. Genomic and vaccine advances should be developed as adjuncts and judged by real-world outcomes that matter to patients and payers. With deliberate investment in validation, workforce capability, data governance, procurement, and materiovigilance, biotechnology can become a practical driver of equitable oral health while delivering more health for more people, earlier and closer to where they live.

## Figures and Tables

**Table 1:** Potential salivary biomarkers for oral health screening.

Condition/Target	Biomarker/Index Test	Matrix	Intended Public-Health Use	Summary Diagnostic Performance *	Regulatory/Implementation Notes
Periodontitis (presence)	aMMP-8 point-of-care oral rinse test (POC-ORT)	Mouth-rinse/saliva	Community/clinic adjunct screening and monitoring; triage for dental exam	Pooled Se 0.63 (95% CI 0.41–0.82), Sp 0.84 (0.65–0.95) (meta-analysis of 6 studies). In a 2025 home-screening model combined with risk factors, Se 94%, Sp 77% [6].	Not a replacement for periodontal exam. False-negatives possible. Use within multi-test pathways; verify local requirements before population deployment [7].
Periodontitis (activity)	IL-6, IL-8 (salivary cytokines)	Saliva	Adjunct risk flag in programs where exams are limited	IL-6 AUC 0.709–0.852; IL-8 AUC 0.671–0.815 [8].	Investigational; not validated as stand-alone tests.
Periodontitis (presence)	Microbiome signatures	Saliva	Exploratory screening; research use	Reviews report “high” accuracy for specific signatures, but subgingival samples outperform saliva for clinical utility [9].	Assay and threshold variability; not yet suitable for population screening.
Caries risk (adults)	Salivary molecules (e.g., mucins, sCD14, IL-2RA/4/13, urease, carbonic anhydrase VI; plus flow/pH/buffering)	Saliva	Risk stratification to target prevention (not diagnosis)	Consistent associations across 16 studies, but no unified thresholds; meta-analysis not feasible due to heterogeneity [10].	Use as part of multifactorial risk tools; not diagnostic of lesions.
Oral cancer (OSCC/HNSCC)	miRNA panels (saliva ± blood)	Saliva (±blood)	High-risk clinic adjunct; case-finding	Pooled Se 78%, Sp 82%	Biomarker sets differ by study; standardization needed before screening.
Oral cancer (OSCC/HNSCC)	mRNA panels (saliva)	Saliva	High-risk clinic adjunct	Pooled Se 91%, Sp 90% [11].	Promising, but heterogeneity and assay access limit field use.
Oral cancer (OSCC/OPSCC)	DNA methylation panels (saliva)	Saliva	High-risk clinic adjunct; case-finding	Meta-analyses indicate higher accuracy than cytokines; pooled Se ~88%, Sp ~89% in recent synthesis; several single gene/combined panel’s report AUC ≥ 0.80 [12].	Strong early evidence; requires standardized protocols and accessible assays.
OPMD (e.g., oral lichen planus)	miRNA panels (saliva)	Saliva	Specialist triage adjunct	Pooled Se 0.80, Sp 0.89, AUC 0.93 for OLP diagnosis [13].	Not generalizable to all OPMDs; research context recommended.

Abbreviations: Se = sensitivity; Sp = specificity; AUC = area under the receiver-operating-characteristic curve; CI = confidence interval; POC = point of care; ORT = oral rinse test; OSCC = oral squamous cell carcinoma; HNSCC = head and neck squamous cell carcinoma; OPSCC = oropharyngeal squamous cell carcinoma; OPMD = oral potentially malignant disorder. * Summary diagnostic performance as reported in published meta-analyses or representative studies.

## Data Availability

No new data were created or analyzed in this study. Data sharing is not applicable to this article.
